# Enhancing the role of MRI in rectal cancer: advances from staging to prognosis prediction

**DOI:** 10.1007/s00330-025-11463-x

**Published:** 2025-03-06

**Authors:** Xiaoling Gong, Zheng Ye, Yu Shen, Bin Song

**Affiliations:** 1 Department of Radiology, West China Hospital of Sichuan University, Chengdu, China; 2 Colorectal Cancer Center, Department of General Surgery, West China Hospital, Sichuan University, Chengdu, People’s Republic of China; 3 Functional and Molecular Imaging Key Laboratory of Sichuan Province, West China Hospital, Sichuan University, Chengdu, China; 4 Department of Radiology, Sanya People’s Hospital, Sanya, China

**Keywords:** Rectal neoplasms, Magnetic resonance imaging, Artificial intelligence, Radiomics, Neoadjuvant therapy

## Abstract

**Abstract:**

Rectal cancer (RC) is one of the major health challenges worldwide. Accurate staging, restaging, invasiveness assessment, and treatment efficacy evaluation are crucial for its clinical management. Magnetic resonance imaging (MRI) plays a significant role in these processes. However, standard MRI techniques, including T2-weighted and diffusion-weighted imaging, have uncertainties in identifying early-stage tumors, high-risk nodules, extramural vascular invasion, and treatment efficacy, potentially leading to inappropriate treatment. Recent advances suggest that the integration of traditional MRI methods, including diffusion-weighted imaging, opposed-phase or contrast-enhanced T1-weighted imaging, as well as emerging synthetic MRI, could address these challenges. Additionally, improvements in imaging technology have spurred research into advanced functional MRI techniques such as diffusion kurtosis imaging and amide proton transfer weighted MRI, yielding promising results in RC assessment. Total neoadjuvant therapy has emerged as a new treatment paradigm for locally advanced RC, with neoadjuvant immunotherapy and chemotherapy offering viable alternatives to neoadjuvant chemoradiotherapy. However, the lack of standards for the early prediction of patient survival and tumor response to neoadjuvant therapy highlights a critical unmet need in matching therapies to suitable patients. Furthermore, organ preservation strategies after neoadjuvant therapy provide personalized options based on tumor response and patient preferences, yet traditional MRI assessments show significant variability. Radiomics and artificial intelligence hold promise for revealing complex patterns in MRI images associated with patient prognosis and treatment response. This review provides an overview of current MRI advancements in RC assessment and emphasizes how future research can refine tailored treatment strategies to improve patient outcomes.

**Key Points:**

***Question*** *The accurate diagnosis of early-stage rectal tumors, high-risk nodules, treatment responses, and the early prediction of patient survival and therapeutic outcomes remain an unmet need*.

***Findings*** *Visual MRI has improved staging, restaging, and invasiveness evaluation. Advanced MRI, radiomics and artificial intelligence provide significant potential for tumor characterization and outcome prediction*.

***Clinical relevance*** *Advances in visual MRI are improving routine imaging protocols and radiomics and artificial intelligence show promise in enhancing treatment decisions through precise tumor characterization and outcome prediction*.

## Introduction

Rectal cancer (RC) has long posed a significant global health challenge [1, 2]. Owing to advances in treatment methods and multidisciplinary management, patient survival and quality of life have significantly improved. For example, patients who achieve a pathological complete response (pCR) after neoadjuvant chemoradiotherapy (NCRT) can now opt for a “watch-and-wait” approach with close monitoring, avoiding surgery-related complications and a decrease in quality of life [3]. In patients with high microsatellite instability, immunotherapy has shown higher rates of good or complete response [4]. Trials on total neoadjuvant therapy are improving treatment compliance, organ preservation, and long-term survival [5]. Neoadjuvant chemotherapy, which selectively omits radiotherapy, provides comparable local control to NCRT with fewer side effects [6]. However, given the significant heterogeneity among RC patients, current clinical practice still faces challenges in further optimizing individualized treatment strategies.

Magnetic resonance imaging (MRI) is a key imaging tool for assessing tumor characteristics and potential biological behavior. Standard rectal MRI includes high-resolution T2-weighted imaging (HR-T2WI) and diffusion-weighted imaging (DWI). HR-T2WI effectively shows the depth of tumor infiltration, and DWI helps distinguish between tumor and nontumor tissues. However, these methods are limited in their ability to identify early tumors, malignant nodules, positive extramural vascular invasion, and minimal residual disease after treatment [7]. Recent studies have revealed the potential of conventional sequences including DWI and contrast-enhanced T1-weighted imaging (CE-T1WI), as well as advanced methods, in staging and restaging RC. Moreover, the current visual MRI technique is insufficient for assessing tumor function and heterogeneity, leading to unmet needs in the precise prediction of both treatment response and long-term outcomes. Quantitative imaging features, such as diffusion kurtosis imaging (DKI), intravoxel incoherent motion (IVIM), amide proton transfer-weighted (APT_w_), and synthetic MRI-derived functional parameters (with illustration of MRI techniques and key parameters provided in Table 1), as well as MRI-derived radiomic and artificial intelligence (AI) features, may provide important insight into tumor function, the microenvironment, and underlying biological behavior. Thus, many studies have investigated their value in RC assessment. However, the existing reviews lack a comprehensive perspective on those advancements and do not provide a concrete analysis of how to optimize patient selection under multimodal therapies [8–10].

**Table 1:** Illustration of advanced MRI techniques and the key parameters

Advanced MRI	Technique overview	Key parameters
Amide proton transfer-weighted (APT_w_) MRI	APT_w_ MRI is a molecular imaging technique that utilizes specific radiofrequency pulses to selectively saturate the amide protons in proteins and peptides and detects the signal changes resulting from the transfer of these protons to nearby water molecules.	APT_w_ signal intensity: It is obtained by calculating the difference in signal intensity at ± 3.5 ppm chemical shift after saturation and expressed as a percentage. This parameter can indirectly reflecting changes in protein and peptide content in the tissue and thus provide information about the altered metabolic state of the tissue.
Diffusion kurtosis Imaging (DKI)	DKI is a diffusion-weighted MRI technique that quantifies the non-Gaussian diffusion behavior of water molecules by analyzing the kurtosis of their diffusion process, and thus provides more detailed information about the microstructure of the tissues.	(1) Mean Diffusivity (MD): It quantifies the average extent to which water molecules diffuse in different directions and reflects changes in the cell density, integrity, and complex fiber arrangement of tissues. (2) Mean Kurtosis (MK): It quantifies the extent to which the diffusion behavior of water molecules deviates from the Gaussian distribution and reflect the complexity and heterogenicity of the tissue.
Intravoxel incoherent motion (IVIM)	IVIM is a diffusion-weighted MRI technique that separates the effects of molecular diffusion and microcirculatory perfusion in tissues by using multiple different b-values acquisition and biexponential model, thus noninvasively providing more comprehensive microstructural information of tissues.	(1) Apparent Diffusion Coefficient (D): It quantifies the pure diffusion behavior of water molecules in tissue and reflects the density and integrity of cells in the tissue. (2) Pseudo-diffusion Coefficient (D*): It quantifies the contribution of microvascular blood flow to the diffusion signal and reflects the microvessel density and hemodynamics in tissues. (3) Perfusion Fraction (*f*): It quantifies the proportion of microvascular blood flow in the overall diffusion signal and reflects the perfusion status within the tissue.
Synthetic MRI	Synthetic MRI is a technique based on mathematical models that combines data from multiple pulse sequences in a single scan to derive various tissue-specific parameters (such as T1, T2, and proton density) and generate images with different contrasts.	(1) T1 value: It reflect the longitudinal relaxation time of the tissue and can be used to assess the hydration and cell density of the tissue. (2) T2 value: It reflect the transverse relaxation time of the tissue and can be used to assess moisture content and structural integrity. (3) Proton density: It represents the number of hydrogen atoms per unit volume and can be used to assess the water content of the tissue.

Therefore, this review aims to summarize recent developments in MRI for RC evaluation, focusing on staging, restaging, invasiveness assessment, response evaluation, and outcome prediction. To maintain focus, this review will concentrate on research related to early and locally advanced rectal cancer, excluding studies on the assessment of distant metastatic disease. Additionally, this review provides a more comprehensive perspective on how to improve future research to enhance the role of MRI in the clinical management of RC.

## Primary staging and invasiveness assessment

### Primary tumor assessment

Carefully distinguishing between T1 and T2 tumors or T2 and early T3 tumors makes sense in avoiding the overtreatment of early-stage tumors [11]. However, HR-T2WI has insufficient resolution in this respect because of the penetration of small vessels into the muscle layers, and the similarities between tumors and the rectal muscularis, as well as between perirectal reactive fibrosis and malignant infiltration [12, 13].

#### Visual interpretation

**Fig. 1:**
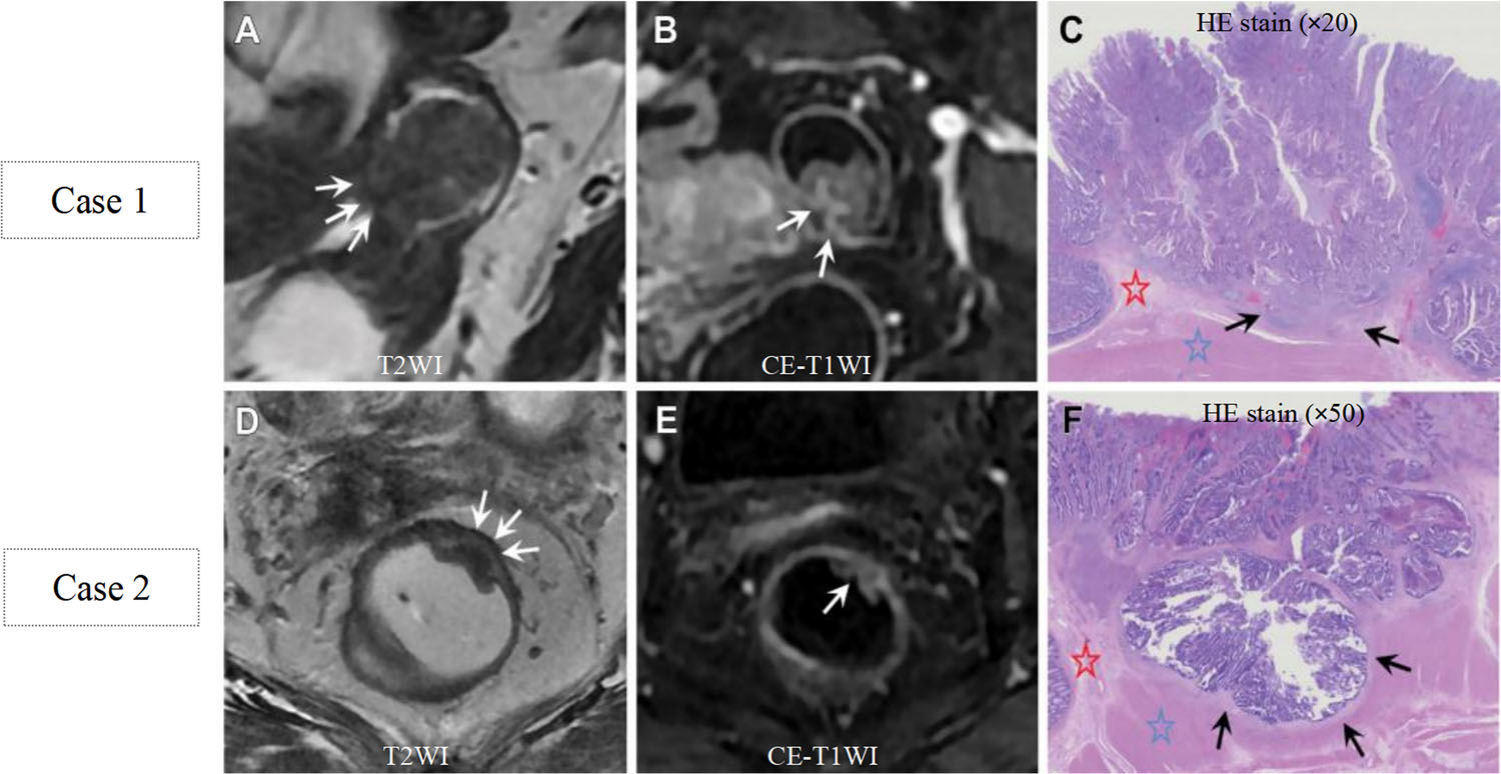
Representative images of pathological T1 (**A**–**C**) and T2 (**D**–**F**) tumors. Case 1: **A** Suspected tumor invasion in muscularis propria and mesorectum (arrows). **B** A continuous and intact submucosal enhancing stripe (SES) (arrows). **C** Tumor invasion into the submucosa (arrows; red star = normal muscularis propria; blue star = submucosa). Case 2: **D** Suspected tumor invasion in submucosal layer and even muscularis propria (arrows). **E** Absence of SES (arrow). **F** Tumor invasion in submucosa and extending into muscularis propria (arrows; red star = submucosa; blue star = muscularis propria). CE-T1WI, contrast-enhanced T1-weighted image; HE, hematoxylin-eosin; T2WI, T2-weighted image. Used with permission of Radiological Society of North America, from [14]; permission conveyed through Copyright Clearance Center, Inc

Histologically, a continuous and intact submucosa indicates that the tumor has not penetrated beyond it, whereas local disruption or complete interruption of the submucosa suggests that the tumor has infiltrated it or even deeper layers. This sign on CE-T1WI could also play an important role in distinguishing T0-1 from T2 tumors (Fig. 1), with an accuracy of 87% [14].

Synthetic double inversion recovery imaging can increase tissue contrast through manual adjustment of the inversion time. Wang et al [15] reported that, on synthetic double inversion recovery images, a “black line”-like opposite magnetization artifact appeared at the junction between tumors and extramural fibrosis. A continuous and smooth “black line”-like artifact could indicate T2 rather than T3 tumors. The integration of this imaging technique with HR-T2WI increased the area under the curve (AUC) for differentiating between T1-2 and T3-4 tumors for both junior (from 0.842 to 0.918) and senior radiologists (from 0.917 to 0.938).

#### Functional parameters

Although previous studies have suggested that the average APT_w_ signal intensity (SI) value of tumors increases with the T stage [16, 17], this finding contradicts the latest findings [18]. This may be related to the different devices used. Moreover, the consensus of the current studies is that T2 values are higher in T1-2 tumors than in T3-4 tumors [15, 19]. This may be attributed to the increased cellularity, nuclear polymorphism, and nuclear/cytoplasmic ratio in T3-4 tumors, resulting in a reduction in the extracellular fluid space. Despite the different cut-off values reported, these studies all suggest that T2 values may help distinguish between T1-2 and T3-4 tumors. Therefore, further research is needed on the value of APT_w_ SI and T2 values in distinguishing among T stages.

However, current research indicates that, when used alone, the mean apparent diffusion coefficient (ADC) value, DKI-derived mean diffusivity (MD), and mean kurtosis (MK) values [16, 20–22], as well as the T1 and proton density values derived from synthetic MRI [15, 19], may be of no value in differentiating between T1-2 and T3-4 tumors.

#### AI and radiomics

A few studies have reported the potential of MRI-based radiomic and deep learning (DL) models in differentiating T1-T2 from T3-T4 tumors [23, 24]. However, even if those models performed well, they did not demonstrate effectiveness in differentiating adjacent stages such as T2 from T3. Separate subanalyses may be recommended in future studies to better capture the subtle differences between these stages.

### Nodal assessment

Solitary nodules in the mesorectum may be normal lymph nodes, metastatic lymph nodes (MLNs), or tumor deposits (TDs). MLNs are confined within or extend beyond the lymph node capsule (extranodal extension, ENE). TDs are localized clusters of cancer cells without identifiable vascular, perineural, or lymphoid structures. Since MLNs and TDs represent a more malignant stage and require additional interventions [25, 26], priority should be given to identifying them. However, the current criteria are unreliable in this area.

#### Visual interpretation

**Fig. 2:**
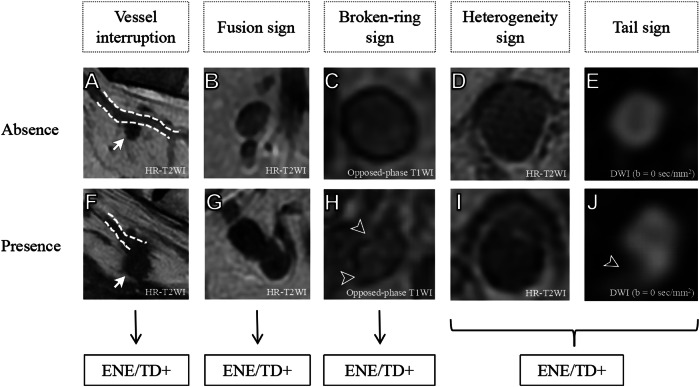
Representative images indicating the absence (**A**–**E**) or presence (**F**–**J**) of extranodal extension or tumor deposits (ENE/TD). **A**, **F** Vessel interruption by nodes: **A** absent, **F** present; dashed lines indicate vessels, arrows indicate nodes. **B**, **G** Fusion of nodes: **B** absent; **G** present. **C**, **H** Broken-ring sign: **C** absent; **H** present; arrowheads in H indicate broken capsule of the node. **D**, **I** Intra-nodal heterogeneity: **D** absent; **I** present. **E**, **J** Tail sign: **E** absent; **J** present; arrowhead indicates tail-like high signal attached to the node. DWI, diffusion-weighted image; HR-T2WI, high-resolution T2-weighted image; T1WI, T1-weighted image. Used with permission of Radiological Society of North America, from [27]; permission conveyed through Copyright Clearance Center, Inc

Li et al [27] reported that the interruption of vessels by nodes or the fusion of nodes on HR-T2WI or a broken capsule on opposed-phase T1WI, as well as the presence of both intranode heterogeneity on HR-T2WI and a tail-like high signal directly attached to the nodes on DWI (*b* = 0 s/mm^2^), were strong indicators of ENE+/TD+ (Fig. 2) [27]. This approach could identify ENE+/TD+ with an AUC of 0.91, a specificity of 94% and a negative predictive value (NPV) of 99% [27]. The validity of these imaging features suggests that they can provide an important complement to the current assessment criteria.

#### Functional parameters

Current studies have revealed that relatively lower T2 values of nodes [28] or primary tumors [29] could help detect MLNs, with AUCs of 0.990 and 0.854, respectively. The higher cellularity within MLNs or in tumors with MLNs may be attributed to this association. In addition, it may be beneficial to consider the relative differences in functional parameters between peri- and intratumor areas or between nodules and tumors [30, 31]. However, research regarding the value of IVIM, DKI, and APT_w_ MRI-derived parameters in identifying MLNs has yielded contradictory results [21, 22, 32–34]. Most of them suggest that the MD [16, 21, 22, 33] and mean APT_w_ SI values [17, 18] of tumors lack discriminatory value, but the higher MK values of tumors might be related to MLNs [16, 21, 22, 33]. Given the heterogeneity in imaging equipment and protocols in current studies, future research should prioritize standardizing imaging in larger-scale studies to further assess the potential applications of those functional parameters in identifying MLNs and TDs.

#### AI and radiomics

Current research on radiomics and AI features derived from lymph nodes for assessing nodule status remains limited in both quantity and quality [35–38]. Extensive manual delineation may have posed a significant challenge to this area. By using a DL tool for automatically detecting and segmenting lymph nodes, Xia et al further developed a weakly supervised model to automatically diagnose MLNs [39]. This model significantly improved the diagnostic speed and accuracy for both junior and experienced radiologists, with AUCs increasing from 0.69 to 0.80 and from 0.79 to 0.88, respectively [39]. However, their reference standard for labeling nodes was based on subjective interpretations by radiologists, which may introduce variability and limit the model’s accuracy. Thus, a one-to-one correspondence between imaging and pathological nodules is essential for constructing high-quality models in future studies.

In addition, some evidence has shown that the tumor and peritumoral features could help to identify nodules [40, 41]. Therefore, further exploration in the field is encouraged.

### Extramural vascular invasion assessment

Positive extramural vascular invasion (EMVI+), defined as the presence of tumor cells in the extramural vessels, provides high-speed passage for potential distant metastasis and is considered one of the high-risk factors for poor prognosis [26, 42]. However, the current standard for diagnosing EMVI+ has a sensitivity of only 62% in distinguishing normal vessels (scores of 0–2) from early EMVI+ (score of 3) [43].

#### Visual interpretation

**Fig. 3:**
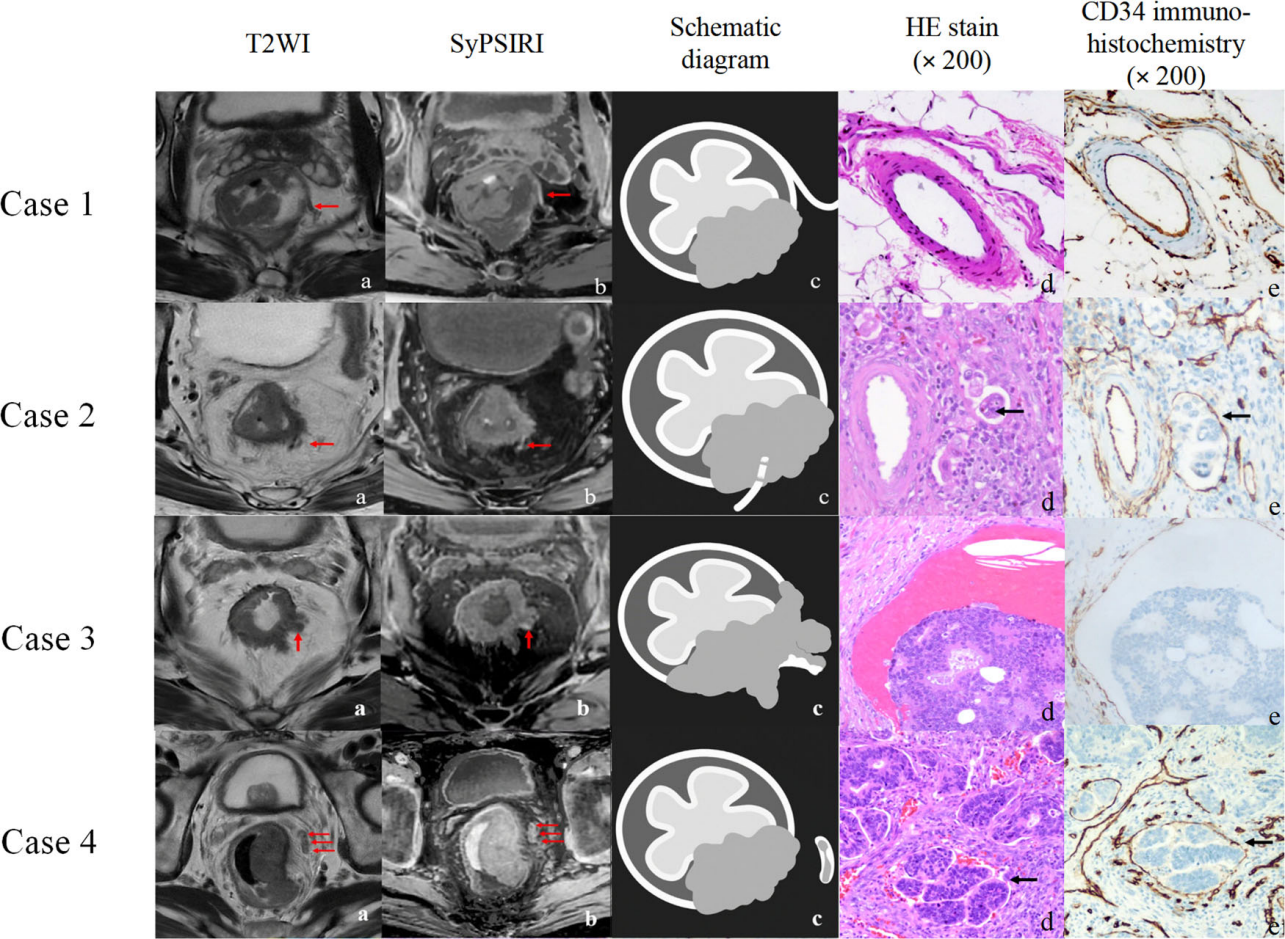
Representative images of the normal extramural vessel (Case 1) and different patterns of extramural vascular invasion (Cases 2–4). Case 1: **a**–**e** Extramural vessel without luminal dilation or tumor invasion (arrows). Case 2: **a**–**e** Extramural vessel with tumor invasion but without luminal dilation (arrows). Case 3: **a**–**e** Extramural vessel with an irregularly dilated lumen and tumor invasion (arrows). Case 4: **a**–**e** Extramural vessel with a uniformly dilated lumen and tumor invasion (arrows). CD34, cluster of differentiation 34; HE, hematoxylin-eosin; SyPSIRI, synthetic phase-sensitive inversion recovery image; T2WI, T2-weighted image. Used with permission of Springer Nature BV, from [44]; permission conveyed through Copyright Clearance Center, Inc

By enhancing the imaging contrast, the emerging synthetic phase-sensitive inversion recovery images show great advantages in distinguishing between isointense tumors and hyperintense vessels (Fig. 3) [44]. When combined with conventional HR-T2WI, the diagnostic sensitivity and accuracy of early EMVI+ significantly increased, even for senior radiologists, from 49% and 78% to 86% and 91%, respectively [44].

#### Functional parameters

Zhou et al reported that EMVI+ tumors presented significantly higher MK values than negative tumors did, and the MK values outperformed the MD and mean ADC values in detecting EMVI+ tumors (AUCs of 0.779, 0.617, and 0.610, respectively) [33]. This aligns with previous studies [16, 45]. Histogram parameters of APT_w_ MRI, such as higher entropy and kurtosis, from tumors could also help identify EMVI+ [46]. These studies imply that EMVI+ may be more likely to occur in tumors with complex microstructures and active proliferation.

#### AI and radiomics

The limited sample sizes in current research on MRI-based radiomics from primary tumors for preoperatively evaluating EMVI have raised significant concerns about the risk of overfitting in these models [47]. Consequently, the effectiveness of radiomics, as well as AI models, in this regard requires further investigation and validation.

## Efficacy evaluation

Patients who respond well to neoadjuvant therapy (NAT) are candidates for organ preservation strategies, including the “watch-and-wait” strategy and local resection. However, restaging HR-T2WI and DWI are limited in differentiating between ypT0-1 and ypT2-4 tumors, as are identifying the pCR in nodes [7].

### Efficacy evaluation of the primary tumor

#### Visual interpretation

Santiago et al introduced a three-layer sign of low-high/moderate-low on HR-T2WI to depict the complete regression of primary tumors [48]. This sign illustrates residual submucosal and extramural fibrosis after treatment, as well as a normal muscle layer or residual mucus lake in between. Although it demonstrated high specificity and NPV in recognizing pCR (0.919 and 0.843, respectively), its sensitivity and positive predictive value (PPV) were relatively low (0.626 and 0.763, respectively) [49]. This finding suggests that it is still insufficient for identifying pCR in primary tumors.

Recent studies supported the claim that DWI was ineffective in enhancing the performance of T2WI in identifying pCR, but only for patients who underwent NCRT [50, 51]. In contrast, for patients receiving neoadjuvant chemotherapy alone, DWI significantly improved the accuracy of T2WI (0.90 vs. 0.83, *p* = 0.03) [52]. The inflammation and fibrosis associated with NCRT may be more pronounced than those associated with neoadjuvant chemotherapy alone [52], leading to decreased recognition of pCR by DWI. These findings suggest that different NAT regimens may require tailored efficacy evaluation approaches.

**Fig. 4:**
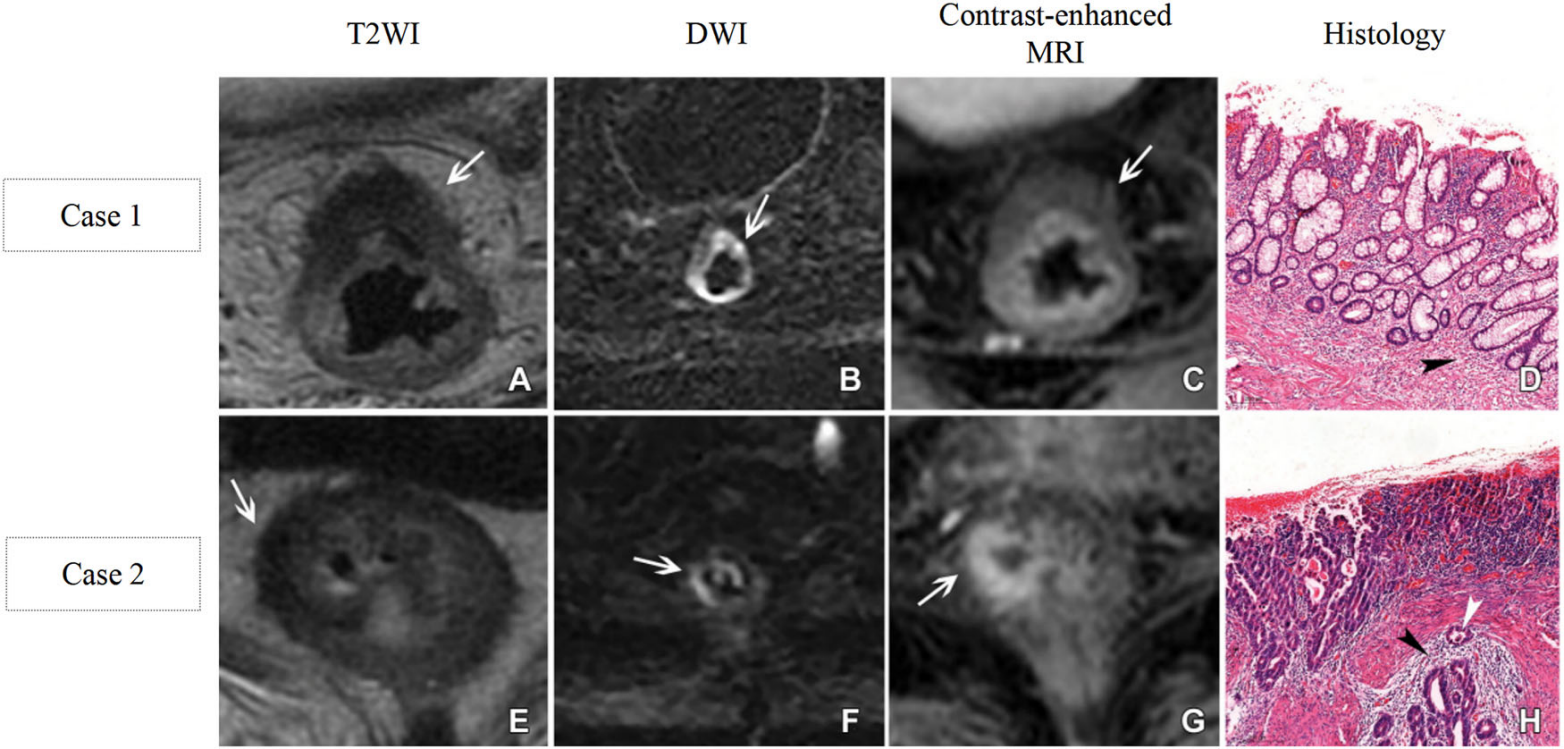
Representative images of lesions with complete response (**A**–**D**) and residual tumor (**E**–**H**) after neoadjuvant chemoradiotherapy. Case 1: **A** Suspicious tumor with low-intermediate signal intensity (SI; arrow). **B** Suspicious tumor with high SI (arrow). **C** No nodular enhancement reaching the outermost edge of the rectal wall (arrow). **D** No residual tumor (arrowhead). Case 2: **E** Suspicious tumor with intermediate-low SI (arrow). **F** No suspicious tumor with high SI (arrow). **G** Nodular enhancement reaching the outermost edge of the rectal wall (arrow). **H** Residual tumor (white arrowhead = residual tumor invasion into the muscularis propria, black arrowhead = fibroblasts surrounding the tumor). CE-T1WI, contrast-enhanced T1-weighted image; DWI, diffusion-weighted image; HE, hematoxylin-eosin; T2WI, T2-weighted image. Used with permission of Radiological Society of North America, from [53]; permission conveyed through Copyright Clearance Center, Inc

**Fig. 5:**
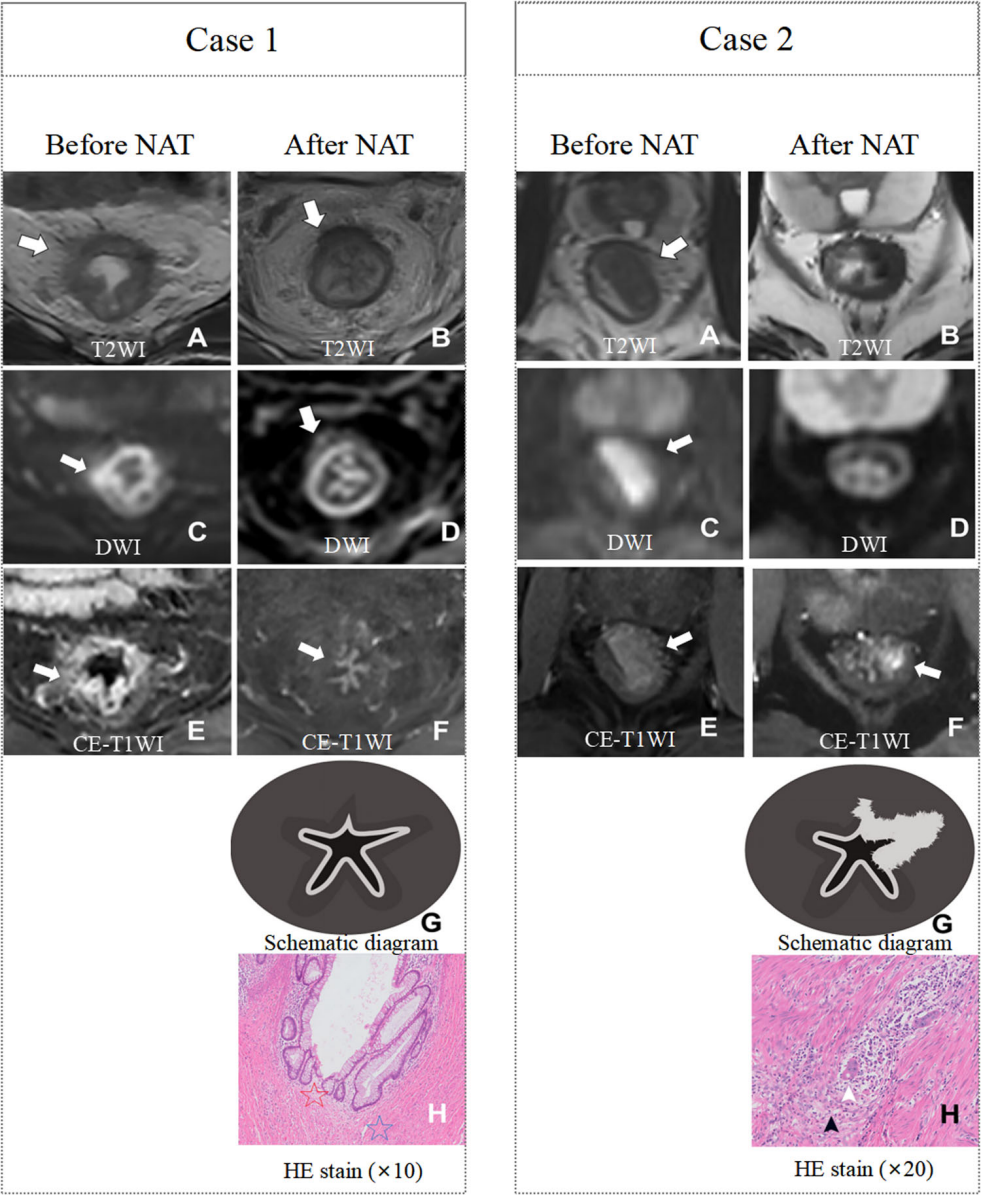
Representative images of patients with complete response (Case 1: **A**–**H**) and residual tumor (Case 2: **A**–**H**) following neoadjuvant chemoradiotherapy. Case 1: **A** Tumor with intermediate signal intensity (SI) (arrow). **C** Tumor with high SI (arrow). **E** Tumor with high SI (arrow). **B** Suspicious tumor with intermediate SI. (arrow). **D** Suspicious tumor with focal high SI (arrow). **F** Mucosal linear enhancement suggesting no residual tumor (arrow). **G** Schematic diagram of mucosal linear enhancement. **H** No residual tumor (red star = intact mucosal layer, blue star = muscularis propria). Case 2: **A** Tumor with intermediate signal intensity (SI) (arrow). **C** Tumor with high SI (arrow). **E** Tumor with high SI (arrow). **B** Suspicious complete response with low SI. **D** Suspicious complete response without high SI. **F** Residual tumor with abnormal nodular enhancement (arrow). **G** Schematic diagram of abnormal nodular enhancement. **H** Residual tumor (white arrowhead = residual tumor invasion into the muscularis propria, black arrowhead = surrounding fibroblasts). CE-T1WI, contrast-enhanced T1-weighted image; DWI, diffusion-weighted image; HE, hematoxylin-eosin; T2WI, T2-weighted image. Used with permission of Radiological Society of North America, from [54]; permission conveyed through Copyright Clearance Center, Inc

In addition, the improvement of CE-T1WI for restaging has also been confirmed. Lu et al [53] reported that a nodular enhancement reaching the outermost edge of the rectal wall on CE-T1WI represents the tumor extending to the rectal muscularis or mesorectum (Fig. 4). Conversely, enhancement within the rectal wall, but not reaching the outermost edge, indicated no or an early-stage tumor (Fig. 4). CE-T1WI outperformed the combination of T2WI and DWI (AUC 0.81 vs. 0.66, *p* < 0.001) in differentiating between ypT0-1 and ypT2-4 tumors [53]. Miao et al [54] reported that mucosal linear enhancement on arterial-phase CE-T1WI represents the absence of residual tumor, whereas abnormal nodular enhancement on arterial-phase CE-T1WI represents the presence of residual tumor (Fig. 5). Moreover, the mucosal linear enhancement on arterial-phase CE-T1WI achieved an AUC of 0.84 in identifying ypT0 tumors [54]. These findings highlight the necessity of adding CE-T1WI to routine restaging MRI.

#### Functional parameters

Previous studies have indicated that the post-treatment mean ADC value and its percentage change demonstrate inadequate accuracy in identifying pCR (74% and 78%, respectively) [55]. The same is true for DKI- and IVIM-derived average parameters and their percentage changes [56, 57].

#### AI and radiomics

Despite the advantages of CT-T1WI in detecting tumor retraction to the mucosa, its effectiveness in differentiating pCR and scattered minimal residual tumor may still be limited. In this context, radiomics analysis may hold some promise.

Recent studies have revealed that radiomics before and after treatment can be used to assess pCR effectively [58–60]. These models significantly outperformed subjective assessments in terms of sensitivity (80% vs. 15.6%, *p* < 0.001) [59] and specificity (90% vs. 85%, *p* < 0.001) [60], as well as overall accuracy. In addition, DL algorithms potentially offer deeper insights than predefined traditional radiomics in response evaluation. Jin et al proposed a deep neural network based on both pre- and post-treatment MRI images to automatically recognize pCR [61]. In the validation cohort, this model achieved a sensitivity of 91.9%, a PPV of 84.2%, a specificity of 93.0%, and an NPV of 96.5%. Furthermore, integrating features derived from advanced functional MRI may further improve efficacy assessment. Zhang et al added DKI to T2WI to develop a DL model [62]. This model performed perfectly in identifying pCR, with a sensitivity of 100%, specificity of 97.3%, PPV of 90%, and NPV of 100%, outperforming both radiologists (AUCs of 0.66 and 0.72, respectively) and DKI-derived average parameters alone (AUC = 0.76). These results highlight the potential of AI models as auxiliary diagnostic tools for assessing pCR. Considering the impressive performance of the above models, prioritizing real-world validation of their effectiveness may promote their clinical translation.

### Efficacy evaluation of the lymph node

One of the contraindications to organ preservation strategies is the presence of residual MLNs and TDs. However, there are currently no reliable criteria for identifying MLNs and TDs on restaging MRI images.

#### Visual interpretation

According to recent research, the size of the largest lymph node (long axis ≥ 5.5 mm or volume ≥ 77 mm³) or the reduction rate in the short-axis diameter (≤ 70%) on restaging HR-T2WI [63, 64], as well as the primary tumor volume on restaging DWI (≥ 0.684 mm³) and its reduction rate (≤ 82.8%) [65], could provide additional value in identifying sustained MLNs. These findings are promising, particularly for nodes that remain ambiguous under current criteria.

#### AI and radiomics

Compared with radiologists, radiomic models focused on lymph nodes showed greater specificity (60.4% vs. 43.4%) and PPV (46.2% vs. 38.8%) in diagnosing pathological MLN+, resulting in improved overall diagnostic accuracy (AUC 0.812 vs. 0.717) [66]. In contrast, those based on the primary tumor showed limited accuracy in predicting pathological MLN+ [66, 67]. However, given the limited research in this area, it is still too early to draw definitive conclusions, and further investigation is needed.

### Efficacy evaluation of EMVI

Persistent EMVI+ after treatment signifies potential resistance to treatment and is associated with more aggressive tumor biology [68]. The residual lesion corresponding to the primary EMVI may mimic the tumor signal, leading to the ambiguity of EMVI on restaging T2WI. In contrast, by detecting restricted diffusion, which indicates the presence of a tumor, DWI demonstrated higher specificity in detecting EMVI+ after NCRT (0.93 vs. 0.67, *p* < 0.001) [69]. This finding is highly consistent with the study by Kim et al [70]. These findings emphasize that when EMVI+ is present on baseline MRI, radiologists should focus particularly on its performance in restaging DWI.

## Response prediction

Predicting potential efficacy before initial treatment could help adjust the NAT strategy. Patients anticipated to achieve a pCR or near pCR with NCRT may transition to total NAT, whereas patients tolerant of NCRT or neoadjuvant immunotherapy may consider participation in clinical trials or direct total mesorectal excision. However, there are currently no established imaging criteria for early response prediction.

### Visual interpretation

Studies have reported that a series of morphological features on baseline MRI, such as a lower one-third tumor location, a nearly round tumor shape, a length greater than 5 cm, a large tumor volume, and clinical T3-4, clinical N+, EMVI+, or positive mesorectal fascia, might indicate an unfavorable response to NCRT [71–75]. However, a reliable system for classifying potential treatment responses has yet to be established.

#### Functional parameters

Several studies have explored the value of functional parameters before treatment in predicting treatment response (detailed information is shown in Table 2) [56, 62, 76–81]. These studies indicated that lower average diffusion metrics, such as MD and mean ADC values, lower mean T1 and T2 values and higher mean APT_w_ SI values of the primary tumor at baseline were associated with a favorable response to NAT. In particular, the mean T2 value predicts pCR, with an AUC of 0.831 [76], and the APT_w_ SI value predicts good response, with an AUC of 0.824 [79]. This may be because tumors with a high proliferative index, adequate tumor vasculature and oxygenation, and increased metabolic activity are more susceptible to chemotherapy and radiotherapy. Therefore, the evaluation based on these functional imaging features may provide valuable evidence for selecting patients who benefit from NAT.

**Table 2:** Research on functional MRI parameters for response prediction before neoadjuvant therapy

Study	Year	Study design	Study center	Sample size	Neoadjuvant therapy	MRI modality	Region of interest	Aim	AUC
Iafrate [81]	2023	Retrospective	1	36	TNT	DWI	Whole tumor	ypT0N0	ADC: 0.797
Lian [76]	2023	Prospective	1	63	NCRT	Synthetic MRI, DWI	Whole tumor	AJCC TRG 0	T1 value: 0.767. T2 value: 0.831. PD or ADC value: NA.
								ypT 0-1	T1 value: 0.746. T2 value: 0.820. PD value: NA. ADC value: 0.612.
Ge [77]	2022	Prospective	1	41	NCRT	T2 mapping	Whole tumor	Mandard TRG 1–2	T2 value: NA.
Li [78]	2022	Prospective	1	79	NCRT	IVIM, DWI	Whole tumor	Mandard TRG 1	D, D*, f, and ADC values: NA.
								Mandard TRG 1–2	D, D*, f, and ADC values: NA.
								ypN 0	D, D*, f, and ADC values: NA.
Chen [79]	2021	Retrospective	1	53	NCRT	APT_w_-MRI, DWI	Whole tumor	AJCC TRG 0-1	APT_w_ SI value: 0.824. ADC value: 0.707.
Li [80]	2021	Retrospective	1	136	NCRT	DKI, DWI	Whole tumor	AJCC TRG 3	MD value: 0.884. MK value: 0.743. ADC value: 0.794
Yang [56]	2021	Prospective	1	42	NCRT	IVIM, DKI, DWI	Whole tumor	AJCC TRG 0	MD, MK, D, D*, f, and ADC values: NA.
								ypT 0-2	MD value: 0.714. MK: 0.686. D, D*, f, and ADC values: NA
Zhang [62]	2020	Prospective	1	383	NCRT	DKI	Whole tumor	ypT0N0	MD value: 0.76. MK value: 0.53.
								AJCC TRG 0-1	MD value: 0.60. MK value: 0.57.
								ypT 0-2	MD value: 0.63. MK value: 0.60.

*ADC* apparent diffusion coefficient, *AJCC* American Joint Committee on Cancer, *APT*_*w*_ amide proton transfer-weighted, *AUC* area under the curve, *D* true diffusion coefficient, D* pseudo-diffusion coefficient, *DKI* diffusion kurtosis imaging, *DWI* diffusion-weighted imaging, *f* perfusion fraction, *IVIM* intravoxel incoherent motion, *MD* mean diffusion, *MK* mean kurtosis, *MRI* magnetic resonance imaging, *NA* not applicable, *NCRT* neoadjuvant chemoradiotherapy, *PD* proton density, *TNT* total neoadjuvant therapy, *TRG* tumor regression grade

#### AI and radiomics

Previous studies have demonstrated that radiomic features of the primary tumor, peritumoral areas, and nodes on baseline MRI are valuable for predicting treatment response (detailed in Table 3) [62, 82–92]. For example, in a study involving 674 RC patients, Song et al developed and validated a radiomic model based on pretreatment T2WI to predict ypT0 disease [87]. Despite a potential risk of misdiagnosis (AUC 0.784), this model achieved an accuracy of 0.926 in independent validation. Similarly, Jayaprakasam et al created a radiomic model utilizing features from mesorectal fat on pretreatment T2WI, which reached an AUC of 0.860 in identifying ypT0N0 [88]. Moreover, radiomic features from both the primary tumor and lymph nodes exhibited strong predictive capabilities for ypN0, yielding AUCs of 0.831 and 0.865, respectively [82, 85]. In addition to these findings, the study by Feng et al highlighted the correlation between nuclear and microenvironmental characteristics in biopsy pathology and ypT0N0 [89]. More importantly, these findings demonstrated that radiomics could provide crucial complementary information. The integrated radiopathomics model achieved an AUC of 0.812, outperforming each individual omics model in a multicenter, prospective observational cohort (all *p* < 0.0001) [89]. Collectively, these findings emphasize the critical value of radiomics, accompanied by multimodal approaches, in advancing treatment response predictions for RC.

**Table 3:** Research on radiomics and artificial intelligence based on MRI for response prediction before neoadjuvant therapy

Study	Year	Study design	Study center	Sample size	Neoadjuvant therapy	MRI modality	Region of interest	Aim	AUC (development/validation)
Wei [82]	2024	Retrospective	2	150	NCRT	T2WI, DWI	Whole tumor	ypN0	Clinical model: 0.687/0.623 T2WI radiomics model: 0.945/0.601 DWI radiomics model: 0.956/0.589
Wang [83]	2024	Retrospective	3	285	NCRT	T2WI, T1WI	Whole tumor	AJCC TRG 0	Clinical model: 0.613/0.381 Radiomic model: 0.979/0.762 Clinical-radiomic model: 0.970/0.810
								cCR^a^	Clinical model: 0.613/0.565 Radiomic model: 0.763/0.715 Clinical-radiomic model: 0.831/0.718
Cicalini [84]	2024	Retrospective	1	35	NCRT	T2WI, ADC	Whole tumor	Mandard TRG 1–2	Metabolomic model: 0.809/NA T2WI Radiomic model: 0.826/NA ADC Radiomic model: 0.807/NA Radiometabolomic model: 0.864/NA
Zhang [85]	2023	Retrospective	1	78	NCRT	T2WI	Nodes (≥ 3 mm)	ypN0	LN morphology model: 0.757/0.823 Radiomics signature: 0.908/0.865 Radiomics nomogram: 0.925/0.918
Jiang [86]	2023	Retrospective	2	127	NCRT	T2WI, DWI	Whole tumor	Mandard TRG 1–2	T2WI radiomics model: 0.91/0.72 DWI radiomics model: 0.88/0.78 Combined radiomics model: 0.99/0.86 Clinical model: 0.77/0.83 Clinical-radiomics model: 0.99/0.94
Song [87]	2022	Retrospective	4	674	NCRT	T2WI	Whole tumor	AJCC TRG 0	Clinical model: 0.705/0.610 T2WI radiomics model: 0.984/0.784 Clinical-radiomic model: 0.989/0.787
Jayaprakasam [88]	2022	Retrospective	1	236	NCRT	T2WI	Whole tumor	ypT0N0	T2WI radiomics model: 0.89/NA
Feng [89]	2022	Retrospective and prospective	4	1033	NCRT	T2WI, CE-T1WI, DWI	Whole tumor	ypT0N0	Pathomics nucleus model^b^: 0.814/0.733 Pathomics microenvironment model^b^: 0.680/0.630 Combined radiomics model^b^: 0.742/0.716 Radiopathomics model^b^: 0.868/0.812
Shaish [90]	2020	Retrospective	2	132	NCRT	T2WI	Whole tumor and mesorectum	AJCC TRG 0	T2WI radiomics rodel: 0.80/NA
								AJCC TRG 0-1	T2WI radiomics rodel: 0.80/NA
Zhang [62]	2020	Prospective	1	383	NCRT	T2WI, DKI	Whole tumor	ypT0M0	Combined radiomics model: 0.997/0.99
								AJCC TRG 0-1	Combined radiomics model: 0.99/0.70
								ypT 0-2	Combined radiomics model: 0.99/0.79
Cui [91]	2019	Retrospective	1	186	NCRT	T2WI, CE-T1WI, ADC	Whole tumor	ypT0	Combined radiomics model: 0.940/0.944 Radiomics nomogram: 0.948/0.966
Zhou [92]	2019	Retrospective	1	425	NCRT	T1WI, T2WI, CE-T1WI, DWI	Whole tumor	AJCC TRG 3	Combined radiomics model: 0.822/0.773 Clinical-radiomic model: 0.843/0.744

*ADC* apparent diffusion coefficient, *AJCC* American Joint Committee on Cancer, *AUC* area under the receiver operating characteristic curve, *CE-T1WI* contrast-enhanced T1-weighted imaging, *DKI* diffusion kurtosis imaging, *DWI* diffusion-weighted imaging, *NA* not applicable, *NCRT* neoadjuvant chemoradiotherapy, *T2WI* T2-weighted imaging, *TRG* tumor regression grade

^a^ No residual tumor on both post-treatment MRI and endoscopy

^b^ Prospective, external validation

### Survival prediction

The traditional tumor node metastasis classification system is limited in its ability to predict outcomes, primarily because it does not adequately capture tumor invasiveness at the macro level. Other key risk factors associated with poor prognosis in RC patients, including the presence of EMVI+, TD+, involvement of the mesorectal fascia, and poor response to NAT, have been identified [93, 94]. However, even among patients with the same high-risk characteristics, there can be significant variations in survival outcomes. This heterogeneity complicates clinical management and underscores the need for further optimization of prognostic assessments.

Numerous studies have highlighted the prognostic value of radiomic and DL features (Table 4) [88, 95–104]. For example, Liu et al reported that the radiomic signature of the primary tumor on preoperative ADC maps was significantly associated with distant metastasis (hazard ratio = 6.109, *p* = 1.73 × 10^−^¹⁷) [103]. Similarly, the more pronounced the risk indicated by the DL model is, the worse the survival [100, 102]. In addition to imaging features from the primary tumor, characteristics of the peritumoral region also have significant prognostic relevance [88, 95]. As a result, models established on those features exhibited strong performance in prognostic prediction and demonstrated significant potential to surpass traditional prognostic methods, which depend on pre- and post-treatment clinical pathological risk factors. In the study by Jiang et al [100], patients who responded well to NCRT could be identified as high risk by DL models, suggesting that they may benefit from additional treatment. In contrast, in the studies by Liu et al [102] and Zhang et al [101], patients who achieved partial response after NCRT could be identified as low risk by DL models, indicating that intensive therapy may not be necessary for them. These findings indicate that baseline radiomic and DL features may provide an important addition to traditional prognostic assessments and assist in improving current clinical decision-making.

**Table 4:** Research on radiomics or artificial intelligence based on MRI for prognostic prediction in before treatment

Study	Year	Study design	Study center	Sample size	MRI modality	Region of interest	Auto-segmentation	Endpoint	Performance^a^ (development/validation)
Xie [104]	2024	Retrospective	3	1024	T2WI, DWI	Whole tumor	No	Disease-free survival	Clinical model: not reported Radiomics model: 0.71/0.71 Combined model: 0.77/0.77
Guo [95]	2024	Retrospective	1	166	T2WI	Whole tumor and mesorectum	No	Death	Intratumoral model^b^: 0.833/0.813 Peritumoral model^b^: 0.824/0.687 The combined model^b^: 0.954/0.821
Mao [96]	2024	Retrospective	1	194	T2WI, ADC, CE-T1WI	Whole tumor	No	Disease-free survival	Radiomics nomogram: 0.76/0.77 Radiomics signature: 0.72/0.73 Clinical model:0.71/0.70
Huang [97]	2024	Retrospective	2	454	T2WI, DWI, CE-T1WI	Whole tumor	No	3-year local recurrence and distant metastasis rate	Radiomic signature^b^: 0.83/0.82. Valentini’s nomogram^b^: 0.68/0.63. Clinical nomogram^b^: 0.74/0.67. Radiomic nomogram^b^: 0.89/0.84.
Zhao [98]	2023	Retrospective	1	230	T2WI	Whole tumor and peritumor region	No	Distant metastasis-free survival	Radiomics model of GTV: 0.722/0.687. Radiomics model of PTV4: 0.750/0.703. Clinical model: 0.659/0.657. Integrated model: 0.831/0.748.
Zhang [99]	2023	Retrospective	1	161	DWI, IVIM, SEM	Whole tumor	No	5-year overall survival	Clinical-radiomics model: 0.819/0.687.
								5-year progression-free survival	Clinical-radiomics model: 0.833/0.815.
Jiang [100]	2023	Retrospective	2	725	T2WI	Single layer	No	Disease-free survival	Deep learning model: 0.62/0.54. Carcinoembryonic antigen: 0.60/0.55 Integrated model: 0.65/0.55.
								Overall survival	Deep learning model: 0.78/0.62. CEA: 0.62/0.53 Integrated model: 0.84/0.67
Zhang [101]	2023	Retrospective	3	800	T2WI, ADC	Whole tumor	No	5-year overall survival	Deep learning model: 0.723/0.692 Clinical model: 0.652/0.679 Integration model: 0.743/0.756 Radiomics model: 0.703/0.600 Valentini’s model: 0.6330.750
								Local recurrence-free survival	Deep learning model: 0.679/0.662 Clinical model: 0.672/0.583 Integration model: 0.723/0.716 Radiomics model: 0.747/0.641 Valentini’s model: 0.617/0.565
								Distant metastasis-free survival	Deep learning model: 0.710/0.670 Clinical model: 0.700/0.677 Integration model: 0.751/0.738 Radiomics model: 0.659/0.635 Valentini’s model: 0.609/0.629
Jayaprakasam [88]	2022	Retrospective	1	236	T2WI	Gross mesorectal fat	No	Local recurrence	Radiomics model^b^: 0.79/NA
								Distant recurrence	Radiomics model^b^: 0.87/NA
Liu [102]	2021	Retrospective	3	235	T2WI, ADC	Whole tumor	No	Distant metastasis-free survival	Deep learning model: 0.851/0.747 Clinical model: 0.714/0.601 Nomogram model: 0.865/0.775
Liu [103]	2020	Retrospective	5	629	T2WI, ADC	Whole tumor	No	Distant metastasis-free survival	Radiomic signature: 0.847/0.809 Valentini’s nomogram: 0.686/0.707 Clinical nomogram: 0.682/0.595 Radiomic nomogram: 0.855/0.848

*ADC* apparent diffusion coefficient, *CE-T1WI* contrast-enhanced T1-weighted imaging, *DWI* diffusion-weighted imaging, *GTV* gross tumor volume, *IVIM* intravoxel incoherent motion, *NA* not applicable, *NCRT* neoadjuvant chemoradiotherapy, *PTV4* volume of 4-mm peritumoral region, *SEM* stretched exponential model, *T2WI* T2-weighted imaging

^a^ Unless otherwise specified, all data are presented as C-index

^b^ Data indicated as area under the receiver operating characteristic curve

### Conclusions and future perspectives

This review summarizes the latest advances in the use of MRI-based semantic features and quantitative features in RC assessment. The current evidence shows that visual evaluation based on DWI, opposed-phase T1WI, CE-T1WI, and synthetic phase-sensitive inversion recovery vessel imaging has great value in staging, restaging, EMVI, and efficacy assessment. Accounting for the effects of NAT regimens when interpreting the complete response of primary tumors after NAT via T2WI and DWI is crucial. These meaningful advances may facilitate improvements in imaging procedures and clinical practice.

Functional parameters, such as T2, APT_w_ SI, and MK values, hold great potential in tumor biology beyond morphological assessment. MRI-based radiomic and AI models have shown great potential in efficacy evaluation, prognosis and response prediction. The integration of radiomics and pathomics further highlights the importance of multidimensional information integration in efficacy prediction. Admittedly, functional parameters, radiomics and AI are not yet ready to be integrated into clinical practice. This may be attributed to differences in data acquisition and processing methods, unclear substitutability of emerging features to traditional features, lack of high-quality validation and biological interpretation, and slow integration of automatic segmentation and diagnostic tools in current research.

**Fig. 6:**
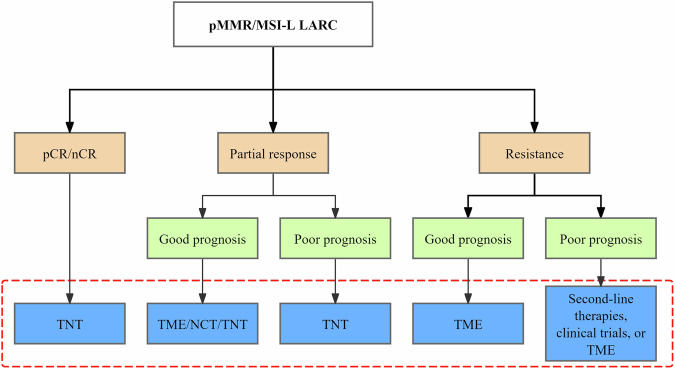
Clinical decisions can be guided through combined efficacy and prognosis predictions. LARC, locally advanced rectal cancer; MSI-L, microsatellite instability-low; nCR, near complete response; NCT, neoadjuvant chemotherapy; pCR, pathologic complete response; pMMR, proficient mismatch repair; TME, total mesorectal excision; TNT, total neoadjuvant therapy

Moreover, a comprehensive perspective could help bridge the translational gap between research and clinical practice, which is currently lacking in existing studies. For efficacy prediction, a major response, including pCR and minimal residual disease in the primary tumor, may widen the number of patients eligible for intensified treatment. Early identification of resistance is equally important for sensitivity prediction, especially for patients considering total neoadjuvant therapy, as timely detection can provide opportunities for treatment adjustment and reduce treatment toxicity. An integrated prediction of both patient prognosis and treatment efficacy may also be valuable in guiding clinical decision-making (Fig. 6). In fact, prognosis does not fully correspond to treatment response. For example, some tumors with potential invasive features may respond well to NAT, whereas some tumors that appear to be low risk may show poor response and undergo rapid progression during NAT. This complexity highlights the importance of comprehensive tumor biology before clinical decisions are made. Finally, given the different tumor response patterns to various treatments, there is also an urgent need to develop predictive models for alternative therapies other than NCRT. Under those potential changes, more patients are expected to benefit from personalized treatment strategies.

## Abbreviations

ADC: Apparent diffusion coefficient
AI: Artificial intelligence
APT_w_: Amide proton transfer-weighted
AUC: Area under the curve
CE-T1WI: Contrast-enhanced T1-weighted imaging
DKI: Diffusion kurtosis imaging
DL: Deep learning
DWI: Diffusion-weighted imaging
EMVI: Extramural vascular invasion
ENE: Extranodal extension
HR-T2WI: High-resolution T2-weighted imaging
IVIM: Intravoxel incoherent motion
MD: Mean diffusivity
MK: Mean kurtosis
MLNs: Metastatic lymph nodes
MRI: Magnetic resonance imaging
NAT: Neoadjuvant therapy
NCRT: Neoadjuvant chemoradiotherapy
NPV: Negative predictive value
pCR: Pathological complete response
PPV: Positive predictive value
RC: Rectal cancer
SI: Signal intensity
TD: Tumor deposit
